# Endoplasmic reticulum stress mediates house dust mite-induced airway epithelial apoptosis and fibrosis

**DOI:** 10.1186/1465-9921-14-141

**Published:** 2013-12-24

**Authors:** Sidra M Hoffman, Jane E Tully, James D Nolin, Karolyn G Lahue, Dylan H Goldman, Nirav Daphtary, Minara Aliyeva, Charles G Irvin, Anne E Dixon, Matthew E Poynter, Vikas Anathy

**Affiliations:** 1 Department of Pathology, Vermont Lung Center University of Vermont College of Medicine, Burlington, VT 05405, USA; 2 Department of Medicine, Vermont Lung Center University of Vermont College of Medicine, Burlington, VT 05405, USA

**Keywords:** Allergen, HDM, Unfolded protein response, ER stress, Apoptosis, Asthma, Airway fibrosis

## Abstract

**Background:**

The endoplasmic reticulum (ER) stress response participates in many chronic inflammatory and autoimmune diseases. In the current study, we sought to examine the contribution of ER stress transducers in the pathogenesis of three principal facets of allergic asthma: inflammation, airway fibrosis, and airways hyperresponsiveness.

**Methods:**

House Dust Mite (HDM) was used as an allergen for *in vitro* and *in vivo* challenge of primary human and murine airway epithelial cells. ER stress transducers were modulated using specific small interfering RNAs (siRNAs) *in vivo*. Inflammation, airway remodeling, and hyperresponsiveness were measured by total bronchoalveolar lavage (BAL) cell counts, determination of collagen, and methacholine responsiveness in mice, respectively.

**Results:**

Challenge of human bronchiolar and nasal epithelial cells with HDM extract induced the ER stress transducer, activating transcription factor 6 α (ATF6α) as well as protein disulfide isomerase, ERp57, in association with activation of caspase-3. SiRNA-mediated knockdown of ATF6α and ERp57 during HDM administration in mice resulted in a decrease in components of HDM-induced ER stress, disulfide mediated oligomerization of Bak, and activation of caspase-3. Furthermore, siRNA-mediated knockdown of ATF6α and ERp57 led to decreased inflammation, airway hyperresponsiveness and airway fibrosis.

**Conclusion:**

Collectively, our work indicates that HDM induces ER stress in airway epithelial cells and that ATF6α and ERp57 play a significant role in the development of cardinal features of allergic airways disease. Inhibition of ER stress responses may provide a potential therapeutic avenue in chronic asthma and sub-epithelial fibrosis associated with loss of lung function.

## Background

Airway inflammation and fibrosis impact lung structure and function in allergic asthma [1]. For instance, chronic asthmatics display extensive airway remodeling characterized by sub-epithelial fibrosis, goblet cell hyperplasia and increased thickness of the basement membrane [2-4]. To date, the processes that facilitate airway fibrosis in allergic asthma remain poorly understood and require a deeper understanding of the cellular and molecular responses to allergens in order to identify potential therapeutic targets.

House Dust Mite (HDM) is one of the most commonly found airborne allergens [5], inducing an allergic response in 50-85% of asthmatics [5,6]. Extracts of HDM contain fungal spores, chitin, fecal pellets (containing proteases), *Dermatophagoide* (Der) family of proteins and lipopolysaccharide (LPS) [7-10]. Studies in rodents have shown that these components can activate multiple receptors present on airway epithelial cells, inducing the secretion of growth factors, the production of cytokines that regulate subsequent activation of T cells, mucus metaplasia, inflammation, airways hyperresponsiveness (AHR), and fibrosis [5,11,12].

Physiological demand for increases in protein folding can create an imbalance in synthesis and capacity to fold. This leads to an increase in misfolded proteins in the endoplasmic reticulum (ER), initiating the ER stress response [13]. In mammalian cells, misfolded proteins are sensed by three ER transmembrane proteins: Inositol Requiring Enzyme 1 (IRE1), activating transcription factor 6 (ATF6), and PKR-like ER kinase (PERK) [14]. A prolonged unfolded protein response (UPR) can cause CCAAT/enhancer-binding protein (C/EBP) homologous protein (CHOP)-induced apoptosis [13]. Additionally, to cope with excessive protein folding load, the protein disulfide isomerases (PDIs), which construct disulfide bridges (−S-S-) in the ER, are upregulated [15]. One such PDI, ERp57, mediates misfolded protein-induced apoptosis by oligomerization of Bak through the formation of inter-molecular disulfide (−S-S-) bridges and the permeabilization of mitochondria [16]. Studies thus far have investigated ER stress-dependent IRE1 signaling during mucus metaplasia in ovalbumin-induced allergic airway disease [17,18]. ER stress is known to play a prominent role in apoptosis of alveolar type II epithelial cells in Idiopathic Pulmonary Fibrosis (IPF) [19,20] and Hermansky Pudlak Syndrome (HPS) [21]. It remains unknown whether ER stress responses are triggered by human asthma relevant allergens such as HDM. Furthermore, it is not clear whether allergen-induced airway epithelial ER stress and apoptosis are linked to sub-epithelial fibrosis and impairment in respiratory mechanics in a murine model of allergic airway disease.

The goal of the present study was to evaluate the impact of HDM, an asthma-relevant allergen, on ER stress responses, apoptosis in airway epithelial cells and subsequent effects on fibrosis and lung function. Our results demonstrate enhanced expression of ER stress transducers in murine and human epithelial cells in response to HDM challenge. In mice, airway epithelial ER stress was associated with up regulation of apoptotic and fibrotic markers after HDM exposure. *In vivo* siRNA mediated knockdown of ATF6α and ERp57 attenuated inflammation and AHR, and abrogated airway fibrosis. These results indicate a critical role of airway epithelial ER stress in allergen-induced airway inflammation and fibrosis.

## Materials and methods

### Cell culture, siRNA transfection and caspase-3 assay

A human bronchial epithelial cell line (HBE) was kindly provided by Dr. Albert van der Vliet-University of Vermont, and cultured as described previously [22,23] and primary human nasal epithelial cells were cultured as described previously [24]. Human cell lines were exposed to either PBS or 25 μg/ml of HDM (Greer, Lenoir, NC). All protocols that utilize primary human nasal epithelial cells were approved by the University of Vermont Institutional Review Board. Cells were transfected with plasmids or siRNA as described [25,26]. Caspase-3 activities were measured using Caspase-Glo 3 (Promega, Madison, WI) reagents, according to the manufacturer’s protocol (Promega, Madison, WI). Results were expressed in Relative Luminescence Units (RLU), after subtraction of background luminescence values. Cell death was measured by MTT assay [25]. All results were obtained from 3 independent experiments conducted in triplicate.

### HDM and OVA-LPS models of allergic airway disease

For all experiments, 8 to 12 wk old WT BALB/c mice (Jackson Laboratories) were used, as approved by the Institutional Animal Care and Use Committee. Mice (n = 10/group) were anesthetized with isofluorane and exposed to 50 μg of the allergen, HDM (GREER-containing 35 endotoxin units/mg) extract, resuspended in PBS, via intranasal administration on day 0 and boosted again on day 7. Mice were then administered 50 μg of HDM consecutively on days 14–18, and euthanized 48 h post final exposure. The control group was given 50 μl of sterile PBS alone at all time points. Alternatively, mice were sensitized via oropharyngeal administration of 100 μg of low endotoxin Ovalbumin (Grade V, Sigma Aldrich) in PBS with 0.1 μg of LPS on days 0 and 7, challenged using 6 doses of aerosolized 1% OVA in PBS for 30 min on days 14–19, and euthanized on day 21. This protocol was adapted from a previously described method of airway sensitization and challenge [27].

### SiRNA administration of ERp57 and ATF6α

Mice (n = 10/group) were anesthetized with isofluorane and administered 10 mg/kg of scrambled small interfering (si) RNA or siRNA for ERp57 (Thermo Scientific-L45187) and ATF6α (ORIGENE-SR418766) oropharyngeally on days −1, 6, and 13, and again on days 16 and 19X. Simultaneously, mice were exposed to 50 μg of HDM resuspended in PBS, or PBS alone via intranasal administration on days 1 and 7. Mice were then administered 50 μg of HDM consecutively, on days 14–18 and euthanized 72 h following the final HDM exposure. On day 16, when siRNA administration coincided with HDM exposure, mice received siRNA 6 h prior to intranasal administration of HDM.

### Assessment of AHR

Mice (n = 10/group) were anesthetized with an intraperitoneal injection of pentobarbital sodium (90 mg/kg), tracheotomized using an 18 gauge cannula, then mechanically ventilated at 200 breaths/min using a FlexiVent™ computer controlled small animal ventilator (SCIREQ). While on the ventilator mice also received the paralytic, pancuronium bromide. The parameters Newtonian resistance (Rn), tissue damping (G), and elastance (H) were calculated as previously described [28,29]. Airway responsiveness is represented as the average of the 3 peak measurements for each animal, obtained at incremental methacholine doses.

### Bronchoalveolar lavage processing

Bronchoalveolar lavage (BAL) from mice (n = 10/group) was collected. Total and differential cell counts were performed as previously described [20]. Briefly, cells were isolated by centrifugation and total cell counts were enumerated using the Advia 120 automated hematology analyzer system. Differential cell counts were obtained via cytospins using Hema3 stain reagents (Fisher Scientific). Differentials were performed on a minimum of 300 cells per animal.

### Western blot analysis

Following dissection, right lung lobes were flash frozen for protein analysis. Lungs were pulverized, and lysed in buffer containing 137 mM Tris∙HCl (pH 8.0), 130 mM NaCl, and 1% NP-40. Proteins from cell lysates were prepared in the same buffer. Insoluble proteins were pelleted via centrifugation, and following protein quantitation of the supernatant, samples were resuspended in loading buffer with dithiothrietol (DTT), and resolved by SDS-PAGE. Proteins were transferred to PVDF and membranes were probed using a standard immunoblotting protocol using the following primary antibodies: P-IRE, IRE, GRP78, ATF6^50^ and CHOP (Abcam), ERp57, GRP94 (Stressgen), Poly (ADP-ribose) polymerase (PARP) (BD Pharmingen) and β-actin (Sigma).

### Non reducing gel electrophoresis

Lung homogenates were resuspended in loading buffer without the reducing agent dithiothrietol (DTT). A separate set of samples were resuspended in loading buffer with DTT to reduce the disulfide bonds. The samples were resolved by SDS-PAGE and subjected to western blot analysis.

### Immunofluorescence

Following euthanization, left lobes were fixed with 4% paraformaldehyde, stored at 4°C overnight for fixation of the tissue, mounted in paraffin, and 5 μm sections were affixed to glass microscope slides for histopathology as previously described [30]. Sections were prepared for immunofluorescence by deparaffinizing with xylene and rehydrating through a series of ethanols [30]. For antigen retrieval, slides were heated for 20 min in 95°C citrate buffer (pH 6.0) with 0.05% TWEEN-20 then rinsed in distilled water. Sections were then blocked for 1 h in 1% bovine serum albumin (BSA) in PBS, followed by incubation with primary antibody for ERp57 (Stressgen), and Caspase-3 (Cell Signal) at 1:500, overnight at 4°C. Slides were then washed 3x5min in PBS, incubated with Alexafluor 647 at 1:1000 in 1% BSA, and counterstained with DAPI in PBS at 1:4000 for nuclear localization. Sections were imaged using a Zeiss 510-META confocal laser scanning microscope.

### Measurement of collagen and immunohistochemistry

Collagen content was measured via the Sircol assay (n = 10/group) (Biocolor Ltd, UK). Briefly, lung lobes were diced and placed in 500 μl of 10 mg/mL pepsin in 0.5 M acetic acid for 3 h at 37°C, or until lungs were completely digested. The digest was spun at 10,000 g for 10 min at room temperature. Fifty microliters of the supernatant was mixed vigorously with 500 μL of sircol dye solution for 30 min and then spun again at 10,000 g for 10 min. Excess dye was decanted off, and the resulting pellet was dissolved in 500 μL of an alkaline solution, 200 μL of which was pipetted in duplicates into a 96 well plate and measured at 540 nm. To evaluate regional changes in alpha-smooth muscle actin (αSMA), fixed sections were prepared for immunostaining by deparaffinizing with xylene and rehydrating through a series of ethanols. For antigen retrieval, slides were heated for 20 min in 95°C citrate buffer (pH 6.0), then rinsed in distilled water. Sections were then blocked for 1 h in blocking serum as per manufacturer’s instructions (Vectastain Alkaline Phosphatase Universal, Vector). Slides were then washed in TBS with 0.1% TWEEN-20 3×5 min, followed by incubation with primary antibody for αSMA (Sigma) overnight at 4°C. Sections were washed again and incubated with a biotinylated universal secondary antibody (Vectastain Alkaline Phosphatase Universal, Vector) for 30 min at room temperature. Slides were washed and incubated with the Vectastain ABC-AP reagent (prepared as per manufacturer’s instructions) for 30 min at room temperature. Sections were then incubated with Vector Red Alkaline Phosphatase Substrate Kit I (Vector) for 10 min at room temperature, rinsed with tap water, and counterstained with Mayer’s Hemotoxylin.

### Statistics

All assays were performed in triplicates. Data were analyzed by one-way analysis of variance (ANOVA) using the Tukey’s test to adjust for multiple comparisons or student’s t test where appropriate. Histopathological scores were analyzed using the Kruskal-Wallis test and Dunn's multiple comparison post hoc tests. Data from multiple experiments were averaged and expressed as mean values ± SEM.

## Results

### HDM induces ER stress and death in human epithelial cells

**Figure 1:**
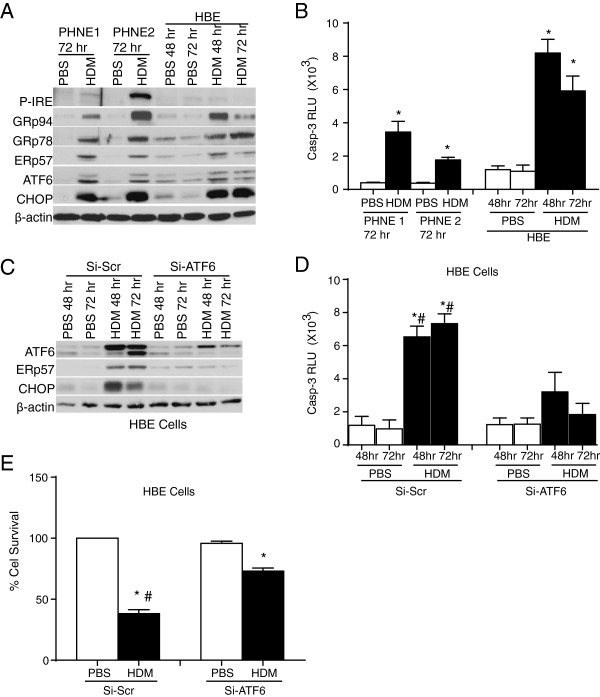
**HDM induces ER stress and activation of caspase-3 in primary human nasal (PHNEs) and bronchial epithelial cells (HBE).** PHNEs from two subjects and HBEs were treated with HDM for the indicated time. The cell lysates were subjected to western blot analysis to detect ER stress markers **(A)**. Caspase-3 activity was measured using a luminescence assay **(B)**. * indicates p < 0.05 as compared to their PBS controls by ANOVA from 2 experiments in triplicate. HBE cells were transfected with siRNA for ATF6α or a non specific Scr sequence, challenged with HDM, and subjected to western blot analysis to detect ER stress markers **(C)**. Caspase-3 activity was measured using a luminescence assay **(D)**. Cell death was quantified using MTT assay **(E)**. * indicates p < 0.05 as compared to their respective PBS controls. # indicates p < 0.05 as compared to their siRNA transfected HDM challenged samples (by ANOVA).

HDM is a complex allergen known to activate multiple receptors and their consequent downstream pathways [5]. In the current study, we hypothesized that these events would result in increased ER stress in epithelial cells. To address this hypothesis, primary human nasal epithelial (PHNE) cells from two non-asthmatic subjects and a human bronchial epithelial (HBE) cell line were challenged with either HDM or PBS as a control. The optimal dose of 25 μg/ml-HDM was selected based on our prior analysis in the laboratory, which showed induction of inflammatory as well as robust ER stress responses in lung epithelial cells (data not shown). Seventy-two hours following repeated challenge, both subjects exhibited increases in phosphorylation of IRE1 (P-IRE), albeit to a greater extent in cells from subject 2, as well as increases in ER chaperone GRP78 (Bip), GRP94, and ERp57. ER stress transducer-ATF6α and downstream transcriptional effector CHOP were also increased after HDM exposure (Figure 1A). With the exception of P-IRE, HBE cells responded in a similar manner with slight differences in kinetics between members of the ER stress responders (Figure 1A). As previously shown, physiological processes demanding a high rate of protein synthesis and secretion may lead to unresolved ER stress resulting in apoptosis [14]. Accordingly, allergen exposure resulted in significant activation of caspase-3 at varying levels in both PHNE and HBE cells (Figure 1B).

During ER stress, activation of ATF6α is known to specifically up regulate PDIs, chaperones, as well as CEBP homologous protein CHOP, and consequently, these events are known to lead to apoptosis [13,31]. To address the contribution of ATF6α activation, chaperone induction, and downstream activation of apoptosis, HBE cells were transfected with either scrambled small interfering (si) RNA (Si-scr) or siRNA for ATF6α. Twenty-four hours following transfection, cells were stimulated with 25 μg of HDM or PBS and harvested at 48 and 72 h after exposure. Knockdown of ATF6α in HBE cells resulted in decreased activation of the 50 kD fragment of ATF6α, CHOP, and ERp57 in whole cell lysates, indicating a requirement for ATF6 in HDM-driven expression of CHOP and ERp57 (Figure 1C). Following HDM administration, active caspase-3 was increased and cell survival was decreased in scrambled siRNA transfected HBE cells. Knockdown of ATF6α in HBE cells showed significant decrease in caspase-3 activity and an increase in cell survival (Figure 1D and E) in cell treated with HDM. These results indicate that allergen (HDM) exposure can induce ER stress, and in turn, lead to apoptosis in human lung epithelial cells.

### HDM induces a robust ER stress response and apoptosis in mouse airway epithelial cells *in vivo*

**Figure 2:**
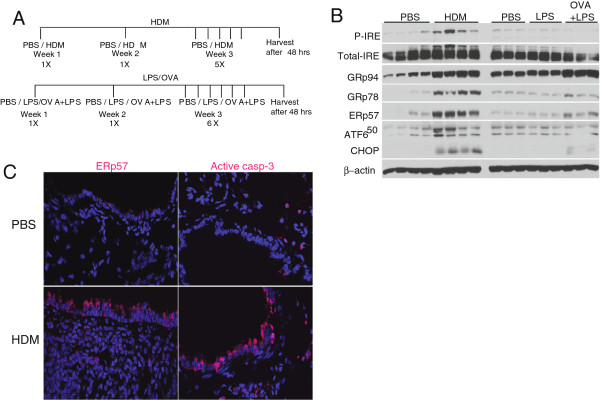
**HDM induces ER stress and active caspase-3.** Mice were challenged with PBS, HDM, LPS or Ovalbumin + LPS as depicted **(A)**. Western blot analysis of whole lung lysates for ER stresses markers **(B)**. Representative images showing up-regulation of ERp57 and active caspase-3 in the airway epithelium of HDM challenged mice (n = 4) **(C)**.

To elicit allergic airways disease we challenged mice with a ubiquitous allergen-HDM, or Ovalbumin and compared the responses with mice treated with LPS (a model of acute lung injury and inflammation). Mice were initially sensitized and challenged via intranasal administration of HDM, LPS or low endotoxin OVA with 0.1 μg of LPS (as an adjuvant) and were euthanized on day 21 [27] (Figure 2A). Results in Figure 2B and Additional file 1: Figure S1 demonstrates activation of ER stress in response to HDM or OVA/LPS in the whole lung, as evidenced by increases in phosphorylation of IRE1, as well as increased expression of GRP78, GRP94 and ERp57. ATF6α and CHOP were also increased after HDM exposure as compared to controls. In contrast to our observation in the HDM model, ATF6α did not appear to increase after OVA/LPS, but we observed a slight elevation in GRP94 and CHOP expression (Figure 2B and Additional file 1: Figure S1). Analysis of inflammatory cells showed a significant increase in eosinophils and lymphocytes in both models as compared to controls (Table 1). Macrophages were decreased in HDM challenged mice as compared to PBS controls, while in LPS and OVA/LPS challenged mice there was a significant increases in macrophages (Table 1). Immunofluorescence of HDM-instilled lungs indicated increased ERp57 as well as active caspase-3 predominantly in the bronchiolar epithelium, as compared to controls (Figure 2C). Collectively these results demonstrate that HDM is a potent activator of ER stress in murine models of allergic airway disease, in comparison to Ovalbumin. Based on these results we continued our evaluations of the role of ER stress in allergic airways disease using the HDM model.

**Table 1:** Inflammatory profiles of models of allergic airway disease as depicted in A

**BAL cell differentials**					
**X10**^**3**^	**PBS**	**HDM**	**PBS**	**LPS**	**OVAL/LPS**
MACS	43.6±5.0	21.4±5.0*	27.2±4.9	40.6±6.6*	100.5±16.4*#
EOS	0.7±0.4	189.4±26.3*	0.0±0.0	0.1±0.1	51.2±10.3*#
PMN	1.3±1.2	1.4±0.2	0.2±0.0	0.1±0.1	21.5±6.0*#
LYMPH	0.7±0.3	15.7±2.1	1.6±0.6	3.0±0.9	29.8±7.1*#

*indicates p<0.05 as compared to their respective PBS controls.

#indicates p<0.05 as compared to their respective LPS treated mice.

### Knockdown of ATF6α and ERp57 decreases CHOP, Bak oligomerization, and apoptosis in mice

**Figure 3:**
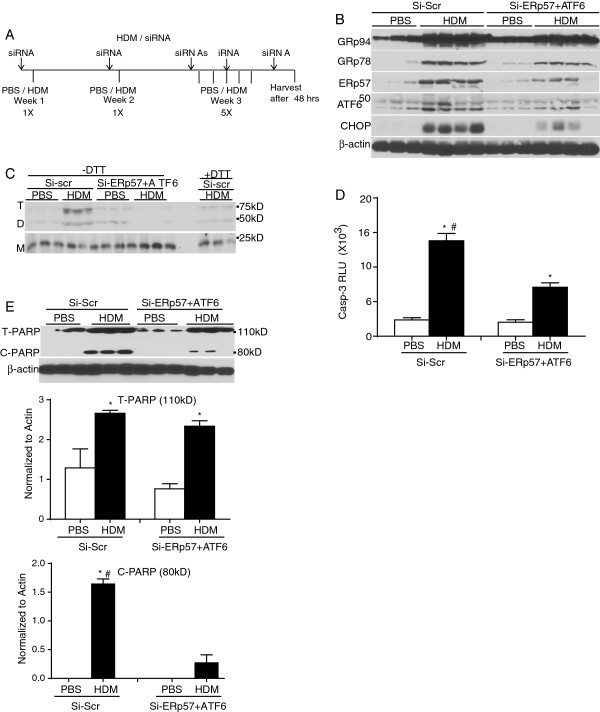
**SiRNA-mediated knockdown of ATF6α and ERp57 decreases -S-S- mediated Bak oligomerization and active caspase-3.** Mice were challenged with HDM and treated with scrambled siRNA (Si-Scr) or ERp57+ ATF6α siRNA (Si-ERp57+ ATF6α) **(A)**. Western blot analysis of whole lung lysates for ER stress markers post siRNA and HDM treatment **(B)**. Disulfide -S-S- mediated oligomerization status of Bak post siRNA treatment (+DTT samples were used as controls) **(C)**. Caspase-3 activity was measured in whole lung lysates using a luminescence assay **(D)**. Cleavage of PARP from whole lung lysates were measured by western blots (T-PARP = total PARP, C-PARP = cleaved PARP) **(E)** and the bar graphs below represent quantitation of total and cleaved PARP by densitometry. * indicates p < 0.05 as compared to their respective PBS controls. # indicates p < 0.05 as compared to their siRNA transfected HDM challenged samples by ANOVA.

To evaluate the role of ATF6α and ERp57 in HDM-induced allergic airways disease *in vivo*, mice were administered 10 mg/kg oropharyngeal scrambled siRNA (Si-scr) or siRNA targeting ERp57 and ATF6α. Subsequently, the mice were challenged via the intranasal administration with 50 μg of HDM consecutively on days 15–19 and euthanized 72 h after final challenge (Figure 3A). As expected, protein expression of ERp57 and ATF6α in the whole lung was decreased after repeated HDM exposure in mice that received siRNA for ERp57 and ATF6α (Si-ERp57 + ATF6) compared to siRNA control samples (Si-scr). We also observed decreases in CHOP and GRP78 in HDM challenged mice treated with Si-ERp57 + ATF6α as compared to Si-scr. The elevated expression of GRP94 was not altered following HDM challenge and treatment with Si-ERp57 + ATF6α relative to Si-scr HDM groups (Figure 3B and Additional file 1: Figure S2), demonstrating that not all ER stress responders were altered by knockdown of ERp57 and ATF6α. ER stress-induced ERp57 is known to form disulfide (−S-S-) bridges in proapoptotic Bak [16]. Results in Figure 3C and Additional file 1: Figure S2 demonstrate that siRNA-mediated knockdown of ERp57 and ATF6α resulted in significantly decreased -S-S- mediated oligomerization of Bak, manifested by decreases in the 75 and 50 kD forms of Bak. Furthermore, we also observed that caspase-3 activity and Poly (ADP-ribose) Polymerase (PARP), a target for caspase-3 cleavage during apoptosis, was significantly decreased in the whole lung lysates after ATF6α and ERp57 knockdown following HDM challenge compared to Si-scr controls (Figure 3D and E). This indicates that knockdown of ATF6α and ERp57 results in significant decrease in HDM-induced, ER stress-mediated apoptotic cascade in the lung.

### Knockdown of ERp57 and ATF6α partially decreases airway inflammation and airway hyperresponsiveness (AHR)

**Figure 4:**
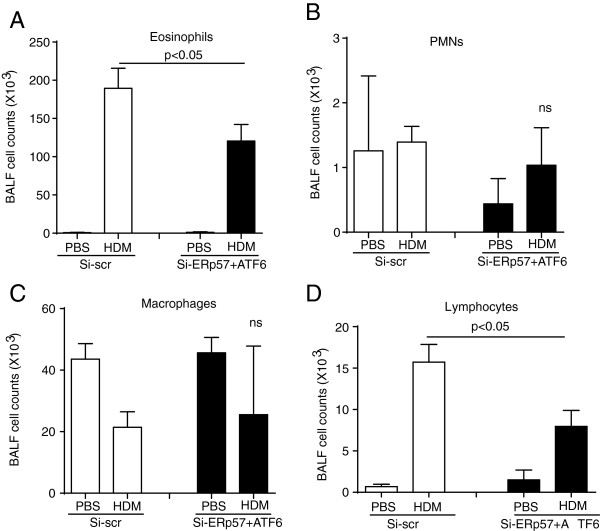
**SiRNA-mediated knockdown of ATF6α and ERp57 partially decreases HDM-induced eosinophils and lymphocytes in BALF.** Mice were challenged with HDM and treated with siRNA as depicted in Figure 3A. BALF fluid was collected from mice (n = 10/group) and eosinophils **(A)**, PMNs **(B)**, macrophages **(C)** and lymphocytes **(D)** were counted using a hemocytometer. p < 0.05 as measured by ANOVA, ns = not significant.

**Figure 5:**
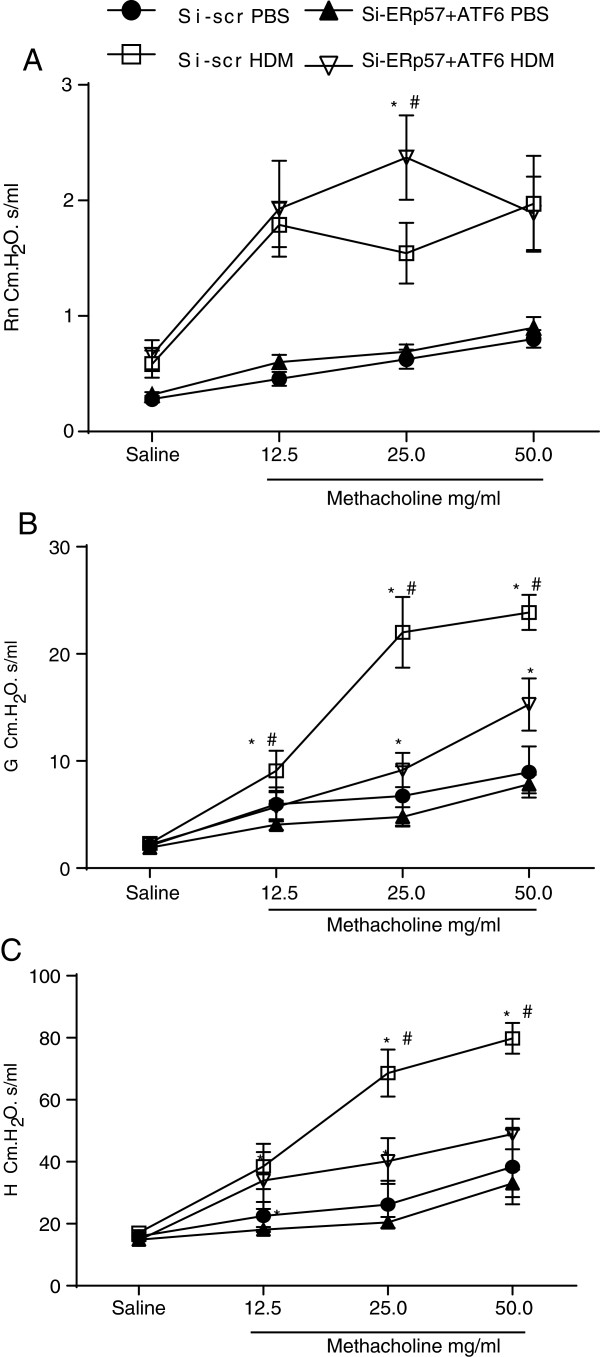
**SiRNA mediated knockdown of ATF6α and ERp57 decreases HDM-induced airway hyperresponsiveness.** Assessment of airway mechanics using a forced oscillation technique. Measurement of Newtonian Resistance (R_n_) **(A)**, airflow heterogeneity or tissue resistance (G) **(B)**, and airway closure/elastance (H) **(C)**, in response to varying doses of methacholine. *p < 0.05 by ANOVA, denotes differences in peak responses, compared with PBS controls. #p < 0.05 by ANOVA, denotes differences in peak responses, compared with the HDM groups (n = 10 mice/group).

As expected, animals in the Si-scr or Si-ERp57 + ATF6 groups that were not exposed to HDM exhibited primarily macrophages in BALF. However, mice that received Si-scr and were challenged with HDM showed a marked influx of cells into the airways, characterized by increases in eosinophils, lymphocytes, and to a lesser extent, neutrophils. In contrast, knockdown of ERp57 and ATF6α resulted in decreases in HDM-induced eosinophils and lymphocytes (Figure 4A, B, C, and D). As expected, we observed significant increases in IL-13 mRNA in HDM challenged mice as compared to PBS controls. Additionally, there was no significant increase in IFNγ mRNA (Additional file 1: Figure S3), indicating that instillation of double stranded siRNA did not alter HDM-induced responses from Th2 to Th1. To address the functional effects of siRNA-mediated knockdown of ERp57 and ATF6α in the airways, a forced oscillation technique was used to evaluate alterations in respiratory mechanics [28,29] in response to HDM challenge. We did not observe any significant differences in central airway resistance (R_n_) at either the lowest or highest dose of methacholine in HDM-challenged siRNA-treated groups of mice, but we did observe significant increases in R_n_ in mice that received Si-ERp57 + ATF6α as compared to Si-scr, after HDM challenge (Figure 5A). In the peripheral airways, tissue resistance/dampening (G) and elastance/stiffness (H) were significantly decreased in mice that received Si-ERp57 + ATF6α as compared to Si-scr, after HDM challenge (Figure 5B and C). Although our analysis revealed an increase in Muc5AC mRNA levels after HDM challenge, there were no significant differences in levels of Muc5AC mRNA or Periodic Acid Schiff (PAS) staining in HDM-challenged ERp57 + ATF6α-siRNA treated groups (data not shown).

### SiRNA mediated knockdown of ERp57 and ATF6α decreases airway fibrosis

**Figure 6:**
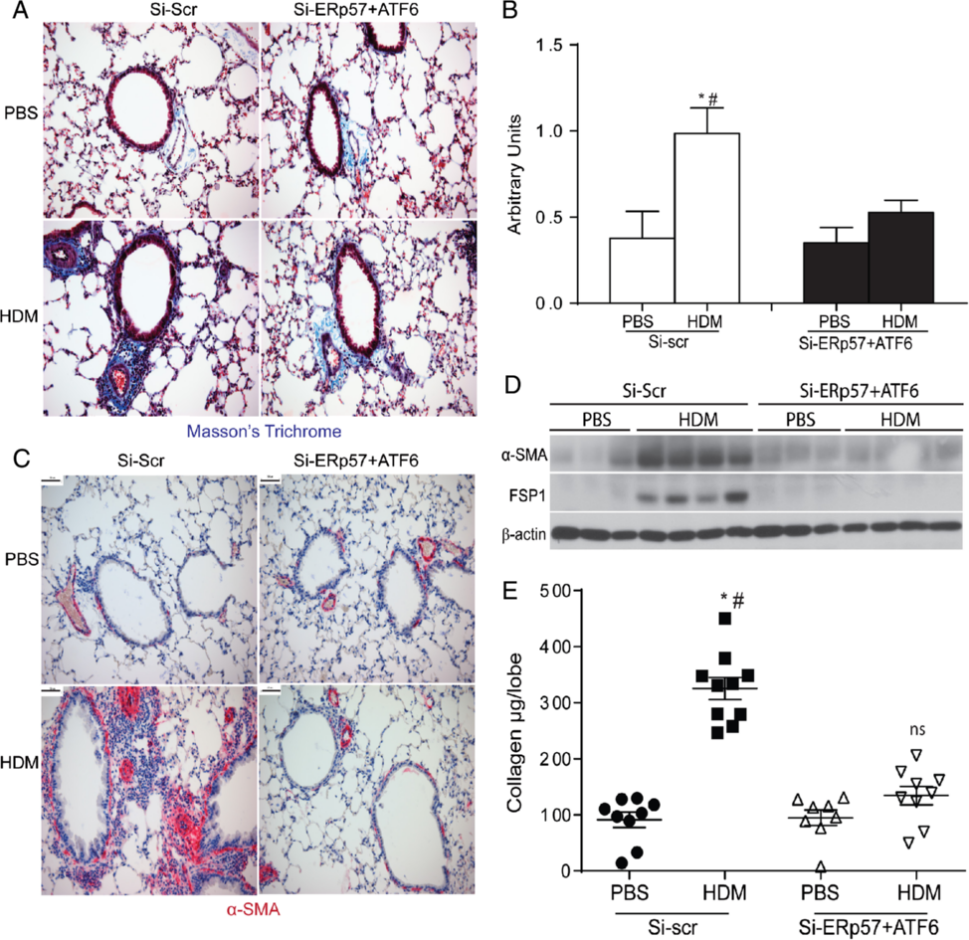
**SiRNA mediated knockdown of ATF6α and ERp57 decreases HDM-induced airway fibrosis.** Representative images and scoring for airways stained with Masson’s trichrome **(A & B)**. * indicates p < 0.05 as compared to their respective PBS controls. # indicates p < 0.05 as compared to their siRNA transfected HDM challenged samples. Representative images stained with an antibody for αSMA **(C)**. Western blot analysis of whole lung lysates for αSMA, FSP1 and β-actin **(D)**. Measurement of collagen **(E)**, * indicates p < 0.05 as compared to their respective PBS controls. # indicates p < 0.05 as compared to their siRNA transfected HDM challenged samples, ns = not significant compared to PBS treated samples.

To examine the role of ATF6α and ERp57 in airway remodeling, collagen deposition was evaluated following siRNA-administration and HDM exposure. Analysis of deposition of collagen by Masson’s Trichrome in mice receiving HDM and Si-scr exhibited significant increases over PBS controls, whereas animals that were treated with HDM and received si-ERp57 + ATF6α showed decreases in Masson’s trichrome staining (Figure 6A) as compared to HDM treated, Si-scr animals. Semi quantitative scoring for Masson’s trichrome staining by three independent scientists blinded to the identity of the samples revealed significant decreases in HDM challenged si-ERp57 + ATF6α treated mice (Figure 6B) over HDM Si-scr treated animals. Additionally, alpha-smooth muscle actin (αSMA) was increased in the peribronchiolar region of HDM challenged mice treated with Si-scr (Figure 6C) as compared to HDM challenged si-ERp57 + ATF6α treated mice. Western blot analysis of whole lung lysates also showed a significant increase in αSMA and Fibroblast Specific Protein (FSP-1) in HDM challenged mice treated with Si-scr (Figure 6D and Additional file 1: Figure S4). These increases were attenuated in mice challenged with HDM and treated with si-ERp57 + ATF6α (Figure 6D and Additional file 1: Figure S4). Furthermore, we also observed an increase in total collagen content in whole lung lysates from HDM challenged mice treated with Si-scr as compared to mice challenged with HDM and treated with si-ERp57 + ATF6α (Figure 6E). Collectively these results indicate that ER stress mediators act to control allergen induced airway fibrosis in the lung.

## Discussion

Perturbations in ER homeostasis can cause ER stress, and when unresolved, ER stress is known to activate cell death [32]. Recent reports suggest that phosphorylation of ER stress transducer IRE1 (P-IRE) and subsequent X-box binding protein 1 (XBP-1) activation is required to induce mucus metaplasia in the lungs of mice challenged with ovalbumin [17,18]. Those reports did not address the implications of ER stress in other facets of asthma, such as epithelial apoptosis, airway hyperresponsiveness and fibrosis. In the current investigation we sought to determine the mechanism by which HDM, a common aeroallergen, induces multiple facets of human asthma in mice. Our results demonstrate induction of severe ER stress in human nasal and bronchial epithelial cells, as well as in mice after administration of HDM. Furthermore, we also found that HDM-induced ER stress is associated with airway epithelial cell death, hyperresponsiveness and subsequent airway fibrosis in mice.

In contrast to recent reports [17,18], our studies with human epithelial cells demonstrated that HDM-mediated activation of IRE1 is not consistent in primary nasal or bronchial epithelial cells. We observed increased phosphorylation of IRE1 in HDM challenged mice as compared to ovalbumin, LPS or ovalbumin/LPS challenged mice. However, we did not observe any downstream XBP-1 activation as in other published reports [17,18]. Activation of PKR-like ER kinase (PERK), phosphorylation of eIF2α, or expression of ATF4, was also not observed. Instead, we showed robust activation of ATF6α and caspase-3 in human epithelial cells and believe that the differences in the activation of specific ER stress transducers may be due to the complex signaling pathways activated in the epithelium by HDM, compared to the antigen ovalbumin or TLR4 agonist, LPS [5,11]. Thus, our results indicate a multifaceted mechanism of allergen-specific activation of ER stress mediators in mouse and human airway epithelial cells.

HDM challenge of human primary nasal and bronchial epithelial cells, as well as HDM exposure of mice, demonstrated consistent robust activation of chaperones, such as GRP94, GRP78 (Bip), ATF6^50kDa^, a PDI-ERp57 and CHOP. Consistent with previously published data, our results demonstrate that chronic ER stress-mediated activation of ATF6α led to increases in specific protein folding enzymes and subsequent activation of apoptotic executioner caspase-3 [14,33,34]. Interestingly, knockdown of ATF6α in human bronchial epithelial cells, as well as ATF6α and ERp57 in mice, resulted in decreased airway epithelial apoptosis as measured by activation of caspase-3. With the exception of GRP78 and CHOP [18], this up-regulation of specific ER stress and pro-apoptotic markers in allergic asthma has not yet been documented. It has been also suggested that chronic activation of ATF6α can lead to the induction of CHOP and subsequent up regulation of proapototic Bak-mediated activation of caspases and apoptosis [16,35-37]. Accordingly, our results suggest that ATF6α activation leads to up regulation of CHOP as well as ERp57, which is capable of inducing disulfide (−S-S-) mediated oligomerization of Bak and induction of intrinsic apoptosis [16].

Our *in vitro* results in human epithelial cells provide a clue that ATF6α as a transcription factor is responsible for ERp57 expression. As shown by others, ATF6α is also known to regulate inflammatory responses in models of other diseases [38,39] alluding to the possibility that ATF6α could be regulating HDM-induced inflammatory responses in the lung.

As reported in neuronal diseases, a severe ER stress response can lead to neuronal cell death [16,40]. Recent studies on ER stress-mediated neuronal apoptosis have also shown involvement of ERp57 in disulfide-mediated oligomerization of proapoptotic Bak [16]. Our work supports the notion that ER stress-induced ERp57 mediates Bak oligomerization and apoptosis of lung epithelial cells during HDM challenge. Furthermore, our results also showed that ERp57 mediated oligomerization of Bak and apoptosis was associated with airway fibrosis. Based on these results, and our data demonstrating ATF6-dependent induction of ERp57, it is reasonable to speculate that ERp57 could be regulating apoptosis of epithelial cells downstream of ATF6 during HDM challenge. However, the role of ATF6α and ERp57 in regulating airway hyperresponsiveness is unknown at this point. Therefore, in the future it would be interesting to conduct careful experiments in mice with lung epithelial cell specific ablation of ERp57 and/or ATF6α to determine the role of these two proteins in allergic airway diseases.

ER stress transducers, such as ATF6 and CHOP, are known to play a prominent role in apoptosis of alveolar type II epithelial cells in fibrotic lung diseases, such as Idiopathic Pulmonary Fibrosis (IPF) [19,20] and Hermansky Pudlak Syndrome (HPS) [21]. Recent studies have suggested that asthmatics and HDM based mouse models of asthma develop sub-epithelial thickening marked by αSMA (smooth muscle hyperplasia) and increased collagen deposition, [2-4] resulting in peribronchiolar fibrosis. Accordingly results presented here show that HDM induces severe ER stress, leading to apoptosis of airway epithelial cells and subsequent fibrosis.

Complex allergens, such as HDM [5], may induce a physiological state that requires an increase in protein synthesis and folding (e.g. production of high levels of mucin, cytokines and surfactants) and create an imbalance in synthesis and capacity to fold, which in turn may increase misfolded proteins in the ER, eliciting the ER stress response and ultimately, apoptosis [15]. Furthermore, the allergen dependent chronic activation of ER stress and apoptosis can cause repeated injury to the airway epithelium. In fact, injured epithelium in human asthmatics as well as in mouse models up regulate profibrotic growth factors, stimulating proliferation of the underlying smooth muscle cells, and subsequently leading to the deposition of extracellular matrix proteins [41]. In our studies, we were unable to observe the up-regulation of various pro-fibrotic growth factors, perhaps as a consequence of the protracted time of *in vivo* analyses.

Eosinophilic inflammation is one of the hallmarks of allergic asthma [42]. In our study, we found that knocking down ATF6α and ERp57 resulted in partial attenuation of eosinophilic influx and lymphocytes in the BALF of HDM-challenged mice, indicating that ER stress may play a role in HDM-induced inflammatory responses. Our results also show that ER stress and apoptosis are associated with another critical facet of asthma, airway hyperresponsiveness to methacholine. HDM-induced tissue resistance (G) and tissue stiffness (H) were decreased in mice after knockdown of ATF6α and ERp57. It is tempting to speculate that these changes in respiratory mechanics are due to altered permeability of the small airways in association with enhanced apoptosis of epithelial cells, which could perhaps allow increased access of methacholine to smooth muscle cells [43,44]. Interestingly we did not observe statistically significant differences in central airway resistance (Rn) in HDM-challenged, Si-scr mice as compared to HDM-challenged, Si-ERp57 + ATF6α mice. The mechanisms of uncoupling of R_n_ in these models are yet to be determined.

## Conclusion

Collectively, our work illuminates a previously unexplored mechanism for HDM-induced airway inflammation and fibrosis via ER stress and apoptosis of epithelial cells. We also show that knocking down ER stress transducers ATF6α and ERp57 decreases HDM-induced apoptosis of airway epithelial cells, bronchiolar fibrosis, inflammation, and airway hyperresponsiveness.

## Abbreviations

HDM: House Dust Mite; OVA: Ovalbumin; LPS: Lipopolysaccharide; UPR: Unfolded protein response; IRE: Inositol requiring enzyme; GRP78: Glucose regulated protein 78; ATF6: Activating transcription factor 6; ERp57: Endoplasmic reticulum protein 57; CHOP: C/EBP homologous protein; PDI: Protein disulfide isomerase.

## Competing interests

The authors declare that they have no competing interests.

## Authors’ contributions

SMH, MEP and VA designed research. SMH, JET, JDN, KGL, DHG, ND, MA and VA conducted experiments. AED provided human epithelial cells and necessary reagents to grow human cells. CGI, MEP and AED provided help with data interpretation. SMH and VA wrote the paper. All authors read and approved the final manuscript.

## Supplementary Material

Additional file 1 Endoplasmic Reticulum Stress Mediates House Dust Mite-induced Airway Epithelial Apoptosis and Fibrosis. Click here for file
