# Supplementary material for: Endoplasmic reticulum stress mediates house dust mite-induced airway epithelial apoptosis and fibrosis
Source: Respir Res. 2013 Dec 24;14(1):141. doi: 10.1186/1465-9921-14-141 (PMC3877992; doi:10.1186/1465-9921-14-141)

**Supplementary Methods**

**RNA isolation and mRNA assessment-** Flash frozen right lobes of lungs were homogenized in liquid nitrogen and RNA was extracted, DNAse treated using RNEasy columns (QIAgen) and reverse transcribed into cDNA using MMLV (Gibco-BRL) according to manufacturer’s instructions. Inflammatory gene expression was determined by semi-quantitative RT-PCR using an Applied Biosystem CFX1000 with 25ng of cDNA per reaction and SYBR green for individual target genes. The following forward and reverse primers were used to amplify the IL-13 (Fwd:5’-CCAGGCCCCTTCTAATGA-3’, Rv:5’-GCCTCTCCCCAGCAAAGTCT-3’) and IFNγ (Fwd:5’-GCGTCATTGAATCACACCTG-3’ Rv:5’-ACCTGTGGGTTGTTGACCTC-3”). The fold induction was normalized using the housekeeping gene cyclophilin.

**Densitometric Analysis:** Densitometric analyses were performed utilizing BioRad® VersaDoc™ Imager Software (Minneapolis, MN). Briefly, Western Blot x-ray films were imaged and rectangles of identical size were traced around each band and assessed for density (intensity/mm^2^). Values were normalized to corresponding β-Actin or total IRE bands following background subtraction.

**Supplementary Figure Legends**

**Figure S1.** Quantitaion of bands on the western blots in figure 2B by densitometry. All samples were normalized to actin except P-IRE.* indicates p<0.05 as compared to PBS controls, # indicates p<0.05 as compared to their LPS and † indicates p<0.05 as compared to LPS/OVA samples by ANOVA. In the bar graph for GRP94 ‡ indicates p<0.05 as compared to HDM samples.

**Figure S2.** Quantitaion of bands in the western blots in figure 3B and 3C by densitometry. All samples were normalized to actin.* indicates p<0.05 as compared to PBS controls, # indicates p<0.05 as compared to their HDM samples and † indicates p<0.05 as compared to DTT treated samples by ANOVA.

**Figure S3.** Measurement of cytokines in the whole lung lysates. IL-13 and IFNγ mRNA levels were measured by qRT-PCR. * indicates p<0.05 as compared to PBS controls, ns-not significant.

**Figure S4.** Quantitaion of bands in the western blots in figure 6D by densitometry. All samples were normalized to actin.* indicates p<0.05 as compared to PBS controls, # indicates p<0.05 as compared to their HDM samples by ANOVA.


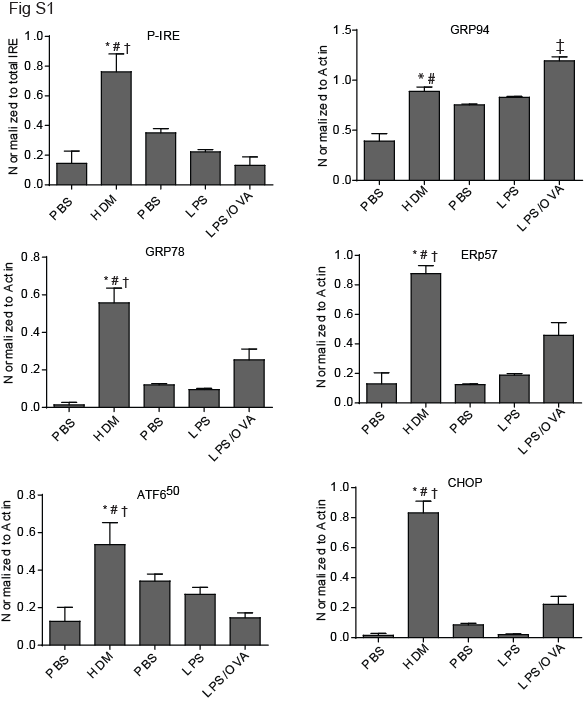


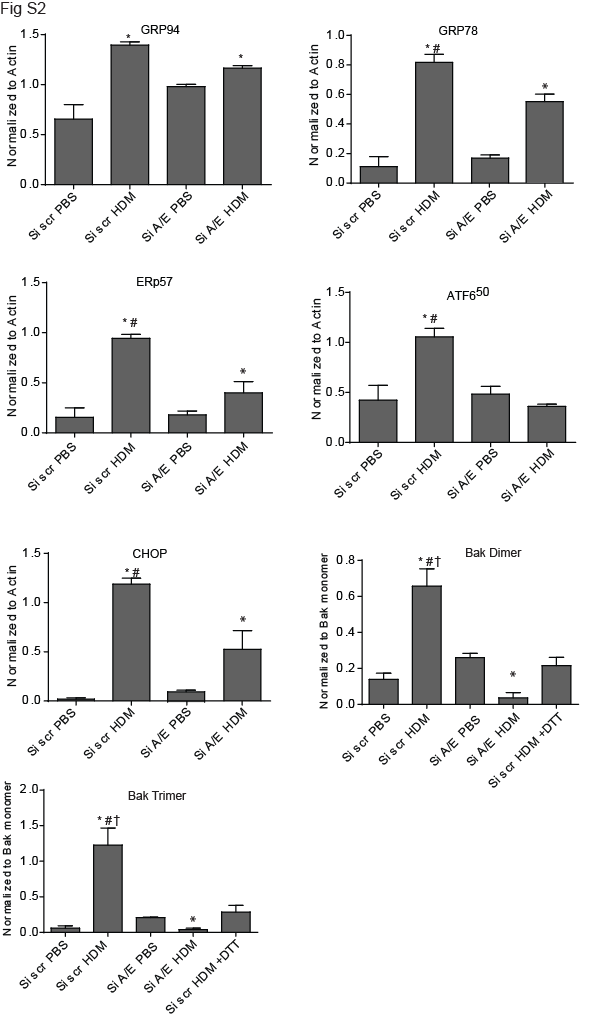


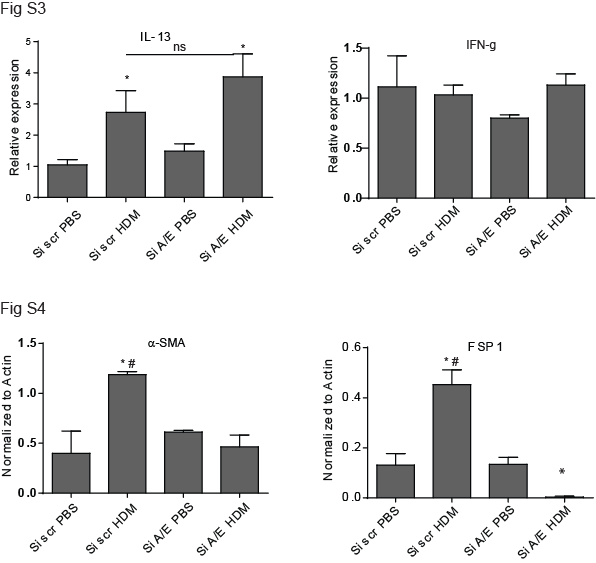

Supplement: Additional file 1 — Endoplasmic Reticulum Stress Mediates House Dust Mite-induced Airway Epithelial Apoptosis and Fibrosis. [file 1465-9921-14-141-S1.docx]
